# SP1-induced long non-coding RNA SNHG6 facilitates the carcinogenesis of chondrosarcoma through inhibiting KLF6 by recruiting EZH2

**DOI:** 10.1038/s41419-020-03352-6

**Published:** 2021-01-11

**Authors:** Fei-Fei Pu, De-Yao Shi, Ting Chen, Yu-Xuan Liu, Bin-Long Zhong, Zhi-Cai Zhang, Wei-Jian Liu, Qiang Wu, Bai-Chuan Wang, Zeng-Wu Shao, Tong-Chuan He, Jian-Xiang Liu

**Affiliations:** 1 Department of Orthopaedics, Union Hospital, Tongji Medical College, Huazhong University of Science and Technology, 430022 Wuhan, Hubei Province P.R. China; 2 No.1 Middle School Attached to Central China Normal University, 430223 Wuhan, Hubei Province P.R. China; 3 Molecular Oncology Laboratory, The University of Chicago Medical Center, 5841 South Maryland Avenue, MC3079, Chicago, IL 60637 USA

**Keywords:** Cell biology, Diseases

## Abstract

Small nucleolar RNA host gene 6 (SNHG6) is a newly discovered long non-coding RNA (lncRNA), while the regulatory mechanism of SNHG6 in chondrosarcoma is largely unknown. Here we found that SNHG6 expression was upregulated and showed positive correlation with the progression of chondrosarcoma. Functional assays demonstrated that SNHG6 was required for the proliferation, migration, and invasion of chondrosarcoma cells. Mechanistic study revealed that SNHG6 could recruit EZH2 and maintain high level of H3K27me3 to repress the transcription of tumor-suppressor genes, including KLF6. KLF6 was found to bind to the promoter region of SP1 and restrained its transcription, while SP1 could be recruited to the promoter region of SNHG6 and promoted its transcription to form a positive loop. In summary, this study reveals that SP1-induced SNHG6 forms a positive loop to facilitate the carcinogenesis of chondrosarcoma through the suppression of KLF6 by recruiting EZH2, which manifests the oncogenic function of SNHG6 in chondrosarcoma.

## Introduction

Chondrosarcoma is the second most common type of primary bone tumors and is derived from cartilage-producing cells^1^. Conventional chondrosarcoma usually affects cartilage cells in femur (thighbone), arm, pelvis, or knee. Cancer statistic data shows that the 10-year survival rate of patient with conventional chondrosarcoma in grade I, II, and III was 83, 64, and 29%, respectively^1^. Routine chemotherapy or radiotherapy show limited effects on treating chondrosarcoma, so surgery is still the most common used option right now. However, the survival rate of chondrosarcoma has not been changed for four decades^2^, and new therapeutic approaches are urgently required for this unmet medical need.

Long non-coding RA (lncRNA) is one family of non-coding RNAs with >200 nucleotides in length that have versatile functions in numerous biological processes, including tumor progression and inhibition^3^. In particular, recent studies have revealed that lncRNAs also participate in the regulation of chondrosarcoma. For example, Xing Bao and his colleagues found that lncRNA HOTAIR was upregulated in chondrosarcoma tissues and showed positive correlation with the tumor stage and poor prognosis, while knockdown of HOTAIR inhibited the growth of chondrosarcoma cells in vitro and in vivo^4^. Shui et al. reported that upregulated lncRNA BCAR4 could promote the proliferation and migration of chondrosarcoma cells through mammalian target of rapamycin signaling^5^. As one of the newly discovered lncRNA, small nucleolar RNA host gene 6 (SNHG6) has been implicated in the modulation of glioma^6^, colorectal cancer^7^, and hepatocellular carcinoma (HCC)^8^. However, the function and regulatory mechanism of SNHG6 in chondrosarcoma is still elusive.

EZH2 is a histone–lysine *N*-methyltransferase enzyme, which is also the catalytic subunit of polycomb repressor complex 2 (PRC2). PRC2 can catalyze the mono-, di-, and tri-methylation of lysine 27 on histone H3 (H3K27), in which tri-methylation at H3K27 (H3K27me3) is associated with transcriptional repression of crucial genes^9^. However, activating mutations of EZH2 have been reported to exist in some tumors and contribute to the tumor progression by inactivating tumor-suppressor genes^10^. Interestingly, one previous study found that nearly 20% of human lncRNAs physically associated with PRC2, which indicated that lncRNAs might generally modulate their target genes by recruiting PRC2 or EZH2 to their promoter regions^11^. For example, lncRNA PCAT1 was found to promote the metastasis of endometrial carcinoma via the downregulation of E-cadherin by EZH2-mediated methylation^12^; Sun et al. found that EZH2 could epigenetically suppress the lncRNA SPRY4-IT1, which thus facilitated the proliferation and metastasis of non-small cell lung cancer^13^. In colorectal cancer, SNHG6 participated in the suppression of pro-apoptosis gene p21 through EZH2, which thus promoted tumor progression^14^. Nevertheless, whether SNHG6 exerted similar function in the development of chondrosarcoma is not clear.

SP1 is one member of a well-studied zinc finger transcription factor family, which is comprised of SP1, Sp2, Sp3, and Sp4. Extensive studies have demonstrated that SP1 is involved in the regulation of various genes that are essential for cell proliferation, differentiation, apoptosis, and carcinogenesis^15^. Mechanistically, SP1 activates the transcription of its target genes via binding on the putative CG-rich motifs in their promoters^16^. In multiple tumors and tumor cell lines, SP1 expression is usually elevated, which is also taken as one negative prognostic marker^17^. In breast cancer cells, Jianguo Hu group reported that SP1-induced lncRNA AGAP2-AS1 enhanced the chemoresistance of breast cancer by epigenetic regulation of MyD88^18^. Jungong Jin and his colleagues identified that SP1-induced lncRNA CASC11 accelerated the tumorigenesis of glioma through targeting FOXK1 via sponging miR-498^19^. One recent report also revealed that SP1 activation contributed to the high expression of SNHG6 in colorectal cancer cells, which further promoted the progression of colorectal cancer^20^. Therefore, we suppose that this regulation work might also exist in chondrosarcoma.

In the present study, we investigated the function and regulatory mechanism of SNHG6 in the carcinogenesis of chondrosarcoma. Our study showed that SNHG6 expression was upregulated in chondrosarcoma tissues and cell lines, which was induced by SP1 activation. SNHG6 suppressed the transcription of tumor-suppressor gene KLF6 through EZH2, which further promoted the tumorigenesis of chondrosarcoma. Although KLF6 had a repressive function on SP1, the inhibition of KLF6 by SNHG6 dampened this effect and further enhanced SP1 expression, and then induced SNHG6 expression. This positive feedback loop facilitated the proliferation, migration, and invasion of chondrosarcoma both in vitro and in vivo. The mechanism revealed here suggested that SP1–SNHG6–EZH2–KLF6 axis contributed to the progression of chondrosarcoma.

## Methods

### Human samples

To compare the differences for two groups of independent samples by unpaired two-tailed *t* test, at least 17 samples are required in each group. To meet this requirement, 30 patients with stage I (*n* = 18) or stage II/III (*n* = 12) chondrosarcoma were enrolled in this study, which was approved by the Research Ethics Committee of Huazhong University of Science and Technology. Written informed consent was obtained from all the enrolled patients. Tumor tissue samples and peritumor tissue samples were obtained from these patients during surgery, which were then immediately fixed for subsequent immunohistochemical (IHC) staining or directly lysed for subsequent RNA extraction and western blot analysis.

### Cell culture and treatment

The chondrosarcoma cell line HCS-2/8 was purchased from Cell Bank of Chinese Academy of Sciences (Shanghai, China), OUMS-27 was purchased from Japanese Collection of Research Bioresources (JCRB) Cell Bank (Tokyo, Japan), and SW1353 and JJ012 were purchased from American Type Cell Culture (ATCC, Manassas, VA, USA). All these cell lines were authenticated by Short Tandem Repeat assay in our laboratory once in a quarter, and they were tested for mycoplasma by PCR monthly. Mycoplasma-free cells were used in the in vitro and in vivo experiments.

HCS-2/8 and OUMS-27 cells were cultured in complete Dulbecco’s modified Eagle medium (DMEM) supplemented with 5 mM glucose and L-glutamine (Gibco, Grand Island, NY, USA), SW1353 cells were maintained in L-15 medium (Gibco, Grand Island, NY, USA), whereas JJ012 cells were grown in RPMI-1640 medium (Gibco, Grand Island, NY, USA). All these mediums were supplemented with 10% fetal bovine serum (FBS, Gibco, Grand Island, NY, USA), 100 μg/ml streptomycin, and 100 U/ml penicillin (Gibco, Grand Island, NY, USA). Cell culture was performed at 37 °C in a humidified incubator containing 5% CO_2_.

### The isolation of human primary chondrocytes

Human primary chondrocytes were isolated from articular cartilage, which was obtained from patients during surgery. All these collections and subsequent experiments were approved by the Research Ethics Committee of Huazhong University of Science and Technology. Briefly, cartilage samples were dissected out and cut into small fragments (about 2 × 2 mm) in a petri-dish using a scalpel blade, which were then washed by phosphate-buffered saline (PBS) twice. Afterwards, the cartilage tissues were transferred to a 250 ml shake flask with 40 ml pronase solution (1 mg/ml in DMEM/F12 1:1, Gibco, Grand Island, NY, USA/Lonza, Basel, Switzerland), which was shaken at 120 rpm at 37 °C for 30 min. Then 40 ml collagenase solution (1 mg/ml in DMEM/F12 1:1, Gibco, Grand Island, NY, USA/Lonza, Basel, Switzerland) was added and the cartilage tissues were digested for another 18–24 h by shaking at 120 rpm at 37 °C. After that, the cartilage matrix was completely digested, and the chondrocytes were detached into the suspension. Then the cell suspension was filtered through a 70-μm cell strainer, centrifuged at 300 × *g* for 10 min, and washed with PBS for two times. After the isolation, the chondrocytes were re-suspended in 20 ml fresh DMEM/Ham’s F-12 (1:1, Gibco, Grand Island, NY, USA) medium containing 10% FBS (Gibco, Grand Island, NY, USA) and counted for cell density. Primary chondrocytes were plated with the initial density of 1.8 × 10^5^/cm^2^ in a 10-cm dish and cultured in an incubator at 37 °C with 5% CO_2_; culture medium was replaced after 2 days for the first time and every 3 or 4 days afterwards.

### Hematoxylin and eosin (H&E) staining of tumor tissues

Mouse metastatic lung tumor tissues were first cut into small pieces about 3 mm^3^, which were then fixed by 4% paraformaldehyde (PFA) (Meilun Bio, Dalian, Liao Ning, China) for overnight. The fixed tissue samples were then embedded in paraffin and sectioned into pieces 5 μm in thickness. Afterwards, the specimen was then dehydrated in increasing concentrations of ethanol and xylol, followed by brief washing and cell nuclei staining with 5% hematoxylin solution (Sigma-Aldrich, USA) for 10 min. After rinsing in distilled water for 5 min, the stained samples were differentiated in 0.1% HCl–ethanol for 30 s. Then they were rinsed in running water for 1 min, blued in PBST (PBS with Tween 20) for 1 min, rinsed in running water for another 1 min, and washed in 95% ethanol for 10 s. Samples were then counterstained with eosin solution (Sigma-Aldrich, USA) for 2 min. After washing and dehydration, H&E-stained the sections were mounted on fluorescence microscopy (Eclipse Ci-E, Nikon) for imaging. Five randomly selected images were captured for each sample.

### Immunohistochemistry

IHC was applied to measure the expression of KLF6 protein in cartilage tissues and chondrosarcoma tissues. In brief, tissue samples were first cut into small pieces approximating 3 mm^3^. Then they were fixed with 4% PFA (Meilun Bio, Dalian, Liao Ning, China) for 6 h. After that, the fixed samples were cryo-protected in 30% sucrose (Sigma-Aldrich, USA) for 24 h. Frozen samples were then sectioned into pieces 5 μm in thickness and then attached onto slides, washed, and incubated with 3% hydrogen peroxide (H_2_O_2_) in methanol for 10 min to block the activity of endogenous peroxidase. Afterwards, these sections were blocked with 5% FBS and then incubated with primary antibody against KLF6 (1:200, Abcam) at 4 °C overnight. After PBS washing for three times, they were treated with horseradish peroxidase (HRP)-conjugated goat anti-rabbit immunoglobulin G (IgG) antibody (1:500, Abcam) for 1 h at room temperature. Then these sections were gently washed by PBS for five times and incubated with DAB substrate for visualization. After washing and dehydration, the sections were sealed by coverslips and subjected to fluorescence microscopy (Eclipse Ci-E, Olympus) for imaging. Five randomly selected images were captured for each sample.

### Tumor development and lung metastasis model in nude mice

Male BALB/C nude mice were ordered from Hunan SJA Laboratory Animal Co., Ltd. at the age of 8 weeks. They were maintained in pathogen-free animal facilities at Huazhong University of Science and Technology with 12 h light–dark cycle. All the experimental protocols were approved by the Animal Ethics Committee (AEC) of Huazhong University of Science and Technology.

To develop the subcutaneous chondrosarcoma and the metastasis of chondrosarcoma, nude mice were randomly allocated to two groups with seven mice per group. We estimated that five mice per group could guarantee the accuracy and stability of results according to previous studies in our laboratory and other groups, and two mice were used as the backup mice in each group. Mice without tumor growth after the inoculation of SW1353 cells were excluded from the final data analysis due to the technical failure of tumor cell injection. Only five mice with normal tumor growth were used in the final data analysis. The investigators were blind to the group allocation of mice when performing the in vitro experiments with tumor samples from mice.

To develop the subcutaneous chondrosarcoma, one group of mice was subcutaneously injected with 5 × 10^6^ shNC (negative control small hairpin RNA (shRNA))-transduced SW1353 cells into the left oxter flank, the other group of mice was subcutaneously injected with 5 × 10^6^ shSNHG6-transduced SW1353 cells in the same location. Before this injection, recipient mice were anesthetized by intraperitoneal injection of avertin (0.25 g/kg). Tumor volume was monitored every 4 days by the measurement with a vernier caliper. Thirty-two days later, the mice were euthanized by CO_2_ inhalation in a chamber and the tumors in site were dissected out to compare their volume.

To observe the metastasis of chondrosarcoma to lung, 5 × 10^5^ SW1353 cells (transfected with shNC or shSNHG6) were injected into the tail veins of nude mice (*n* = 5). The mice were sacrificed by CO_2_ inhalation at 30 days, and the lung tissues were obtained and photographed. The number of pulmonary nodules was counted. Then the lung tissues were subjected to H&E staining to reflect the pulmonary metastasis.

### Western blotting

SW1353 and HCS2/8 cells were collected and directly lysed in RIPA buffer supplemented with a protease inhibitor cocktail (Sigma-Aldrich, USA). Cell lysates were then quantified with a bicinchoninic acid (BCA) kit (Thermo Fisher Scientific, USA). Twenty micrograms of protein was loaded for each sample and separated by sodium dodecyl sulfate (SDS)–polyacrylamide gel electrophoresis and then transferred to polyvinylidene difluoride (PVDF) membrane (Millipore Corp, Bedford, MA). After blocking with 5% bovine serum albumin in Tris-buffered saline with 0.1% Tween 20 (TBST) for 1 h at room temperature, the PVDF membrane was rinsed and probed with the primary antibodies against the indicated target proteins at 4 °C overnight: anti-HuR antibody (1:1000, Santa Cruz-sc-5261), anti-EZH2 antibody (1:1000, Abcam-ab186006), anti-G9a antibody (1:1000, Abcam-ab40542), anti-p21 antibody (1:2000, Abcam-ab109520), anti-SP1 antibody (1:1000, Abcam-ab124804), and anti-β-actin antibody (1:5000, CST-#4970). After vigorous washing, PVDF membranes were incubated with HRP-conjugated goat anti-mouse IgG (1:5000, Abcam-ab205719) or goat anti-rabbit IgG antibody (1:5000, Abcam-ab205718) for 1 h at room temperature. After washing, the PVDF membranes were incubated with ECL substrate to develop chemiluminescence on blots, which were then captured by the ChemiDoc MP imaging system (Bio-Rad, USA) and quantified by the Image J software (NIH, Bethesda, MD, USA).

### RNA extraction, reverse-transcription, and quantitative real-time PCR (qRT-PCR)

Total RNA was extracted from tissues or cell lines using the RNeasy Mini Kit (Qiagen, Hilden, Germany) according to the manufacturer’s instructions, which were then used for the first-strand cDNA generation with RT2 First Strand Kit (Qiagen, Hilden, Germany). qPCR experiment was performed on Mastercycler® RealPlex^2^ qPCR system (Eppendorf, Hamburg, Germany). The mRNA expression of the target genes was normalized to that of glyceraldehyde 3-phosphate dehydrogenase mRNA. All samples were prepared in triplicate. The primers used in qPCR are listed in Table 1.

**Table 1:** The primers used in quantitative real-time PCR.

Primer name	Sequence (5′–3′)
SNHG6 forward	ATACTTCTGCTTCGTTACCT
SNHG6 reverse	CTCATTTTCATCATTTGCT
P53 forward	CAGCACATGACGGAGGTTGT
P53 reverse	TCATCCAAATACTCCACACGC
P21 forward	TGTCCGTCAGAACCCATGC
P21 reverse	AAAGTCGAAGTTCCATCGCTC
KLF6 forward	GGCAACAGACCTGCCTAGAG
KLF6 reverse	CTCCCGAGCCAGAATGATTTT
LATS2 forward	ACCCCAAAGTTCGGACCTTAT
LATS2 reverse	CATTTGCCGGTTCACTTCTGC
SP1 forward	TGGCAGCAGTACCAATGGC
SP1 reverse	CCAGGTAGTCCTGTCAGAACTT
EZH2 forward	AATCAGAGTACATGCGACTGAGA
EZH2 reverse	GCTGTATCCTTCGCTGTTTCC
GAPDH forward	GGACACAATGGATTGCAAGG
GAPDH reverse	TAACCACTGCTCCACTCTGG

### Plasmid construction and transfection

Endogenous gene knockdown was implemented by shRNA. shRNAs targeting SNHG6, SP1, and KLF6 (shSNHG6, shSP1, and shKLF6) and shNC were purchased from GenePharma (Shanghai, China) and the sequences are listed in Table 2. shSNHG6, shSP1, shKLF6 and shNC were then subcloned into pLKO.1-puro vector (Sigma-Aldrich, US). Sequence-verified shRNA plasmids were then used for lentivirus package in HEK293T cells. Titer-determined lentivirus was then used to infect SW1353 and HCS2/8 cells to obtain stable SNHG6-, SP1-, or KLF6-knocking down cells and negative control cells.

**Table 2:** shRNA sequences used in plasmid construction.

shRNA name	Sequence (5′–3′)
shSNHG6	CCGGCTGCGAGGTGCAAGAAAGCCTCTCGAGAGGCTTTCTTGCACCTCGCAGTTTTT
shSP1	CCGGGCTGGTGGTGATGGAATACATCTCGAGATGTATTCCATCACCACCAGCTTTTT
shKLF6	CCGGCCGTATGATGAGGCCAACTTTCTCGAGAAAGTTGGCCTCATCATACGGTTTTT
shNC	CCGGCCTAAGGTTAAGTCGCCCTCGCTCGAGCGAGGGCGACTTAACCTTAGGTTTTT

Gene overexpression was carried out with lentivirus. Human SNHG6 and KLF6 genes were amplified by PCR from cDNA of SW1353 cells with the primers listed in Table 3. SNHG6 and KLF6 genes were then subcloned into the multiple cloning sites (EcoR I/BamH I) of pLenti-P2A-Puro expression plasmid (OriGene, Rockville, MD, USA), which were then transfected into 293T cells by lipofectamine 3000 (Invitrogen, Grand Island, NY, USA) together with packaging plasmid mixture (OriGene, Rockville, MD, USA) to produce SNHG6- or KLF6-overexpressing lentivirus. Titer-determined lentivirus was then used to infect SW1353 and HCS2/8 cells to generate stable SNHG6- or KLF6-overexpressing cells by puromycin selection.

**Table 3:** Primers used in PCR.

Name	Sequence (5′–3′)
SNHG6 forward	AAAAAAGAATTCCTTTCCCGCGCGACCGGCGAGGG
SNHG6 reverse	AAAAAAGGATCCTTTTTTTTTTTTTTTTTGTAAGG
KLF6 forward	AAAAAAGAATTCATGGACGTGCTCCCCATGTGCAGC
KLF6 reverse	AAAAAAGGATCCTCAGAGGTGCCTCTTCATGTGCAGGGC

### Cell proliferation by MTT (3-[4,5-dimethylthiazol-2-yl]-2,5 diphenyl tetrazolium bromide) assay

SW1353 and HCS2/8 cells were seeded in 96-well plates at a density of 1 × 10^4^ cells per well in 100 µl fresh complete medium (L-15 for SW1353, DMEM for HCS2/8). For each treatment, five repeated wells and one blank control well with culture medium only were prepared. Cells were then incubated at 37 °C in a humidified incubator with 5% CO_2_. Cell viability was monitored using an MTT Assay Kit (Abcam, UK) at 0, 24, 48, 72, and 96 h following the manufacturer’s instructions. Data were collected from four independent experiments.

### Cell colony-formation assay

Twelve-well plate was precoated with a layer of solidified medium containing 0.8% agarose, then 4 × 10^3^ SW1353 or HCS2/8 cells were plated on this layer with 1.5 ml culture medium supplemented with 0.4% agarose. Two weeks later, cell colonies were fixed by 4% PFA and then stained with 0.1% crystal violet solution for 10 min. After washing, the whole well was captured and the number of viable colonies >0.1 mm were calculated using the Image J software (NIH, Bethesda, MD, USA). This experiment was repeated for three times.

### Cell migration and invasion by transwell assay

SW1353 or HCS2/8 cells were detached by trypsinization. After centrifugation and aspiration of the supernatant, the pelleted cells were resuspended and the cell density was adjusted to 1 × 10^6^/ml with serum-free medium (L-15 for SW1353, DMEM for HCS2/8). Then 100 μl cell solution was seeded on top of filter membrane in the Transwell insert (Corning, USA) and incubated in a cell culture incubator for 10 min. To perform the cell invasion assay, the filter membrane in the Transwell insert was precoated with 50 μl Matrigel, which will be solidified in a 37 °C incubator for 30 min, and subsequently, 100 μl cell solution was added on the surface of Matrigel. After that, 600 μl of complete medium (L-15 for SW1353, DMEM for HCS2/8) with 20% FBS was carefully added into the bottom of the lower chamber in a 24-well plate as a chemoattractant. The transwell plate was then cultured for 20 h. Next, the transwell insert was taken out and immersed in 70% ethanol for 10 min to fix the cells, which were then stained by 0.2% crystal violet for 10 min. After gentle washing in distilled water for five times, the cells that have migrated through the membrane toward the chemoattractant and attached on the underside of the membrane were viewed and captured by an IX71 inverted microscope (Olympus, Japan), and six random fields per well were captured. Cell number per well was calculated with the captured images using the Image J software (NIH, USA).

### RNA pull-down assay

SNHG6 and control lncRNA that was complementary to SNHG6 was synthesized by GenePharma Co., Ltd. (Shanghai, China). 3′-Untranslated region of androgen receptor (AR) RNA was used as a positive control for HuR protein due to the UC-rich region of HuR in AR RNA; poly(A)_25_ RNA that did not enrich HuR RNA-binding protein (RBP) was used as the negative control. The RNA pull-down assay was conducted using a Pierce™ Magnetic RNA-Protein Pull-Down Kit (Thermo Fisher Scientific, USA) according to the manufacturer’s manual. Briefly, 1 × 10^7^ SW1353 or HCS2/8 cells were collected and lysed in 1 ml RIPA buffer, which was supplemented with RNase inhibitor (Selleck, USA) and complete protease inhibitor (Selleck, USA). Then, 3 μg testing and control RNA were biotinylated with the Pierce RNA 3′ Desthiobiotinylation Kit. Afterwards, 50 pmol biotinylated RNA was incubated with 50 µl of streptavidin magnetic beads for 15–30 min at room temperature with agitation. After washing with 20 mM TBS, streptavidin magnetic beads were re-suspended in 100 μl 1× Protein-RNA Binding Buffer and mixed with 20 μl cell lysates, which was rotated for 30–60 min at 4 °C. After three rounds of washing, the RBPs were eluted by adding 50 μl Elution Buffer to the beads and incubating for 30 min at 37 °C with agitation. The eluted samples were collected for subsequent western blotting experiments.

### Chromatin immunoprecipitation (ChIP)

After the transfection of shNC and shSNHG6, SW1353 or HCS2/8 cells were harvested, washed, and fixed with 4% PFA for 15 min. Then the fixation solution was removed, and the cells were incubated with 0.65 M glycine solution for 5 min at room temperature with gentle agitation. After that, the fixed cells were washed by PBS and lysed in ChIP lysis buffer (50 mM HEPES-KOH (pH7.5), 140 mM NaCl, 1 mM EDTA (pH8), 1% Triton X-100, 0.1% sodium deoxycholate, 0.1% SDS, 1× protease inhibitors) on ice for 15 min. Afterwards, the lysate was sonicated to shear chromatin DNA into 200–800-bp fragments. After centrifugation, the supernatant was collected and the chromatin fragments were incubated with anti-EZH2 (5 μg, Abcam-ab186006), anti-H3K27me3 (5 μg, Abcam-ab6002), anti-SP1 (5 μg, Abcam-ab231778) primary antibodies or recombinant rabbit IgG (5 μg, Abcam-172730) at 4 °C overnight. Pre-blocked protein A/G beads (by salmon sperm DNA, Thermo Fisher Scientific, USA) were then added to capture immunoprecipitants at 4 °C for 2 h with rotation. After centrifugation, the supernatant was discarded, and the precipitated DNA was eluted by loading 100 μl elution buffer to the protein A/G beads and vertexing slowly for 15 min at 30 °C. The eluted DNA was analyzed by qPCR to detect a 380-bp fragment in the promoter region of p21 and a 390-bp fragment in the promoter region of KLF6 with the following primers: p21-F, 5′-GCTCATTCTAACAGTGCTGTG-3′; p21-R, 5′-CAAGGAACTGACTTCGGCAG-3′. KLF6-F 5′-CCTTTCTCCACTCCAGACTCA-3′; KLF6-R, 5′-GTGTTTATCTTGTAGAGGGCGA-3′. DNA captured by EZH2 and H3K27me3 antibody was normalized to normal mouse IgG preciptated DNA.

### Dual-luciferase reporting assay

The promoter region for the transcription of SNHG6 and SP1 was cloned and inserted into the upstream of luciferase 2 gene in pGL4.17 vector, which was transfected into SW1353 and HCS2/8 cells together with pGL4.73 *Renilla* luciferase control plasmid by lipofectamine 2000 (Thermo Fisher Scientific, USA). The *Renilla* luciferase provided normalization reference. Forty-eight hours after transfection, the SW1353 and HCS2/8 cells were lysed and subjected for luciferase activity measurement with the Dual Luciferase Assay Kit (Promega, Wisconsin, USA) according to the manufacturer’s instructions. Firefly and *Renilla* luciferase activities were determined by a plate reader (NEO, Bio-Tek, USA) and normalized to *Renilla* luciferase data.

### Data analysis

Each experiment was repeated for at least three times, and one representative experiment was shown. Data are shown as mean ± standard deviation (SD), which was analyzed by GraphPad Prism 6 (GraphPad Software, Inc.). The variance of data was estimated for each group in every experiment, which was similar for subsequent statistical comparison. Unpaired two-tailed Student’s *t* test was used to compare the difference between two groups. One-way analysis of variance (ANOVA) followed by Tukey post hoc test was used for multiple comparison. Statistical significance was determined as indicated in the figure legends. Asterisks *, **, and *** denoted significance at 0.05, 0.01, and 0.001, respectively.

## Results

### The expression of SNHG6 in chondrosarcoma tissues and cell lines is upregulated and is associated with clinical classification

**Fig. 1:**
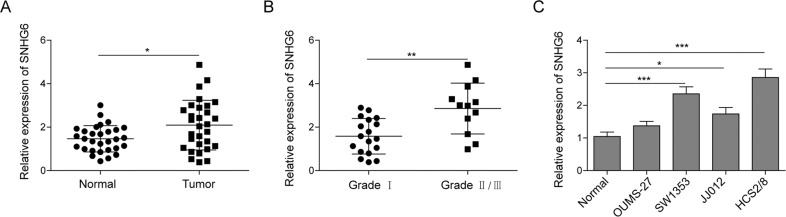
The expression of SNHG6 in chondrosarcoma tissues and cell lines is upregulated and is associated with clinical classification. Chondrosarcoma tissues and normal cartilage tissues (*n* = 30) were collected from patients after surgery. Then the relative expression of SNHG6 in chondrosarcoma tissues and normal cartilage tissues (**A**) or in grade I and grade II/III chondrosarcoma tissues (**B**) was measured by qRT-PCR. **C** The relative expression of SNHG6 in normal chondrocytes and chondrosarcoma cell lines was determined by qRT-PCR. The results represented one of the three independent experiments. Data were depicted as mean ± SD. *p* values were determined by unpaired two-tailed Student’s *t* test (**A**, **B**) or one-way analysis of variance (ANOVA) followed by Tukey post hoc test. ***p < 0.001, **p < 0.01, *p < 0.05.

To investigate the association between SNHG6 expression and the progression of chondrosarcoma, we first measured the expression of SNHG6 in human chondrosarcoma tissues and normal cartilage tissues by qRT-PCR. The data showed that SNHG6 expression was elevated in chondrosarcoma tissues when compared with normal cartilage tissues (Fig. 1A). Moreover, we further analyzed the expression level of SNHG6 in chondrosarcoma tissues in different stages. To our surprise, the chondrosarcoma in stage II/III had significantly higher level of SNHG6 than those in stage I (Fig. 1B), which suggested that SNHG6 expression was positively correlated with the progression of chondrosarcoma. To verify this phenotype, we then assessed the expression of SNHG6 in normal chondrocytes and several available chondrosarcoma cell lines by qRT-PCR, including OUMS-27, SW1353, JJ012, and HCS2/8. Consistently, all kinds of chondrosarcoma cell lines exhibited upregulated SNHG6 expression, in which HCS2/8 showed the highest expression of SNHG6, followed by SW1353 (Fig. 1C). In addition, we measured the migration ability of these cell lines via transwell assay, and the results showed that SW1353, JJ012, and HCS2/8 cells had relatively higher migration ability than that of OUMS-27 cells and normal chondrocytes (Fig. S1). Thus we used HCS2/8 and SW1353 cells in the subsequent experiments. Taken together, these experiments demonstrated that SNHG6 expression was higher in both chondrosarcoma tissues and cell lines than normal tissues and chondrocytes, and its expression showed positive correlation with the migration of chondrosarcoma cells and the clinical progression of chondrosarcoma.

### SNHG6 knockdown inhibits the proliferation, migration, tumorigenesis, and metastasis of chondrosarcoma cells

**Fig. 2:**
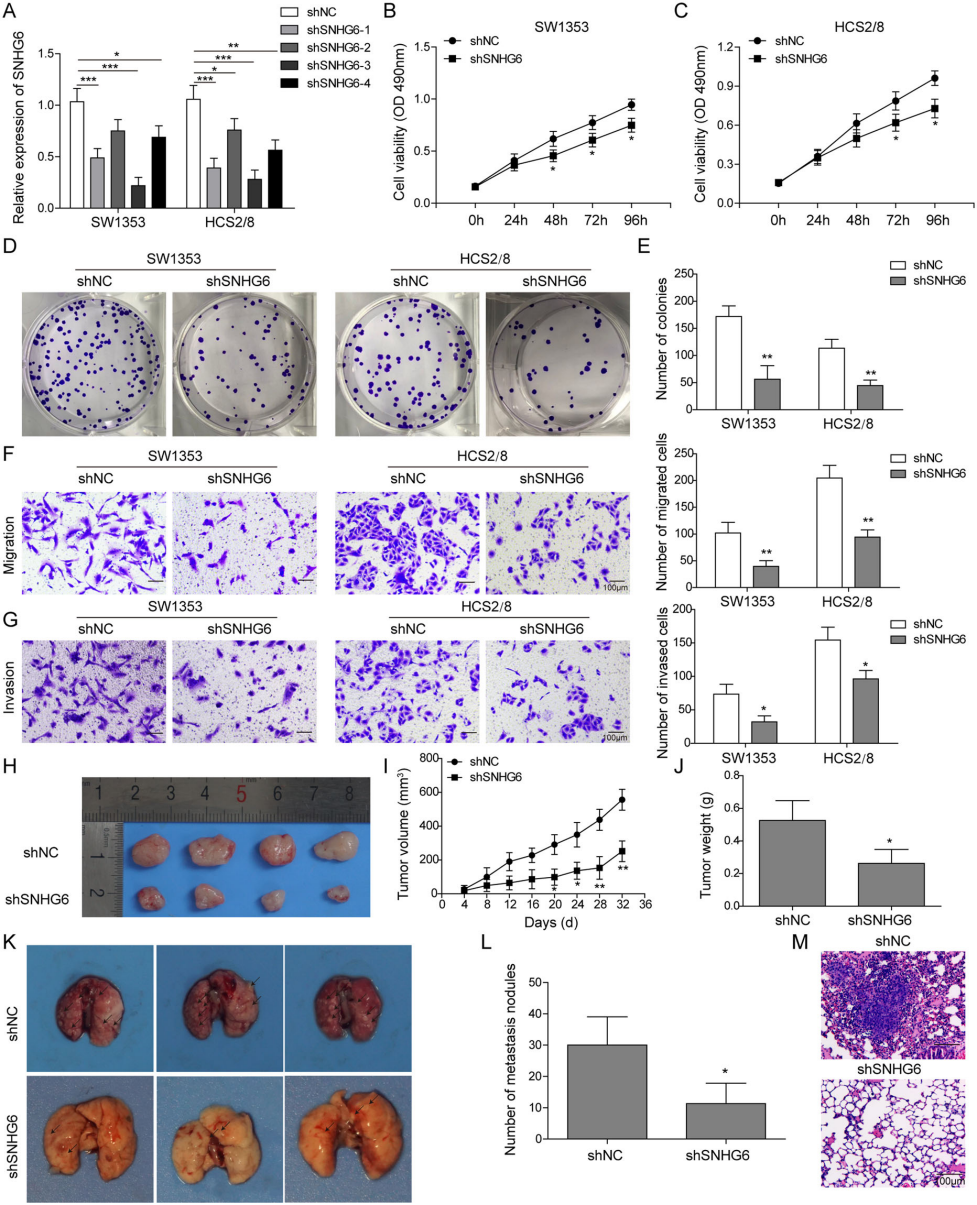
SNHG6 knockdown inhibits the proliferation, migration, tumorigenesis, and metastasis of chondrosarcoma cells. SW1353 and HCS2/8 cells were transfected with control shRNA (shNC) or various shRNAs targeting SNHG6 (shSNHG6) by lentivirus, respectively. **A** The relative expression of SNHG6 in shNC or shSNHG6-transduced SW1353 and HCS2/8 cells was determined by qRT-PCR. SNHG6 RNA level was normalized to GAPDH mRNA; experiments were performed in triple. **B**, **C** The proliferation of SW1353 (**B**) and HCS2/8 (**C**) cells that were transfected with shNC or shSNHG6 was assessed by MTT assay. **D**, **E** Colony-formation assay was performed to examine the influence of SNHG6 knockdown on the proliferation of SW1353 (**D**) and HCS2/8 (**E**) cells. **F**, **G** The migration and invasion of SW1353 (**F**) and HCS2/8 (**G**) cells after the transfection of shNC or shSNHG6 were measured by transwell assay. Left, the representative images of cells that migrated to the bottom well and were stained with crystal violet, scale bar: 10 μm; right, the statistic of cell invasion and migration in transwell assay. **H**–**L** ShNC or shSNHG6-transduced SW1353 cells were subcutaneously injected into male nude mice (*n* = 4). **H** The representative image of grafting tumor tissues that were harvested from sacrificed mice at 32 days after the subcutaneous injection, scale bar: 10 mm. **I** The tumor size was monitored by vernier caliper measurement every 4 days. **J** The weight of tumor tissues that were harvested from sacrificed mice at 32 days after the injection. **K** The representative image of metastatic tumor tissues on the lungs that were obtained from sacrificed mice at 30 days after tail veil injection, scale bar: 10 mm. **L** The statistic of metastatic nodules on the lungs. **M** The invasion of SW1353 cells to lungs was assessed by H&E staining, scale bar: 100 μm. **A**–**M** The results represented one of the three independent experiments. **A**–**C**, **E**–**G**, **H**, **J**, **L** Data are represented as mean ± SD. *p* values were determined by unpaired two-tailed Student’s *t* test (**E**–**G**, **J**, **L**) and one-way analysis of variance (ANOVA) followed by Tukey post hoc test (**A**) or two-way ANOVA (**B**, **C**, **H**). ***p < 0.001, **p < 0.01, *p < 0.05.

To explore the function of SNHG6 in the progression of chondrosarcoma, we then knocked down SNHG6 by shRNA in both HCS2/8 and SW1353 cells and assessed the influence of SNHG6 knockdown on the proliferation, migration, tumorigenesis, and metastasis of chondrosarcoma cells. First, we designed four shRNAs targeting SNHG6 and one shNC and then transduced them into SW1353 and HCS2/8 cells, respectively. The subsequent qRT-PCR experiment demonstrated that all four shRNAs displayed various knockdown efficiencies, in which SNHG6-3 silenced target gene >70% (Fig. 2A). Thus we mainly used this shRNA in the following studies. Next, we compared the cell viability and proliferation in the presence or absence of SNHG6. The MTT assay proved that SNHG6 knockdown significantly impaired the viability of SW1353 and HCS2/8 cells, especially at 72 and 96 h after the assay initiation (Fig. 2B, C); meanwhile, both SW1353 and HCS2/8 cells transfected with shSNHG6-3 showed much less colonies then the control cells (Fig. 2D, E), which further suggested that SNHG6 was required for the proliferation of chondrosarcoma cells. Moreover, we checked the influence of SNHG6 knockdown on the migration and invasion of SW1353 and HCS2/8 cells by transwell assay; consistently, both experiments showed that SNHG6 knockdown restrained the migration and invasion of chondrosarcoma cells (Fig. 2F, G). To confirm these phenotypes in vivo, we then subcutaneously injected ShNC or shSNHG6-transduced SW1353 cells into male nude mice and then measured the tumor volume every 4 days. SNHG6 knockdown significantly reduced the tumor growth, and the tumor volume plus tumor weight were much smaller than the control mice (Fig. 2H–J). In addition, we examined the metastatic tumor tissues on the lungs by tail injection of chondrosarcoma cells, and the data elucidated that SNHG6-silenced SW1353 cells had relatively lower metastatic modules than control cells (Fig. 2K, L). H&E staining with lung tissues further displayed that there were much more infiltration and invasion of chondrosarcoma cells in the lungs when SNHG6 is normally expressed (Fig. 2M). In all, these data demonstrated that SNHG6 knockdown could dampen the proliferation, migration, tumorigenesis, and metastasis of chondrosarcoma cells in vitro and in vivo.

### SNHG6 restrains the expression of tumor-suppressor genes through recruiting EZH2 and maintaining the histone methylation

**Fig. 3:**
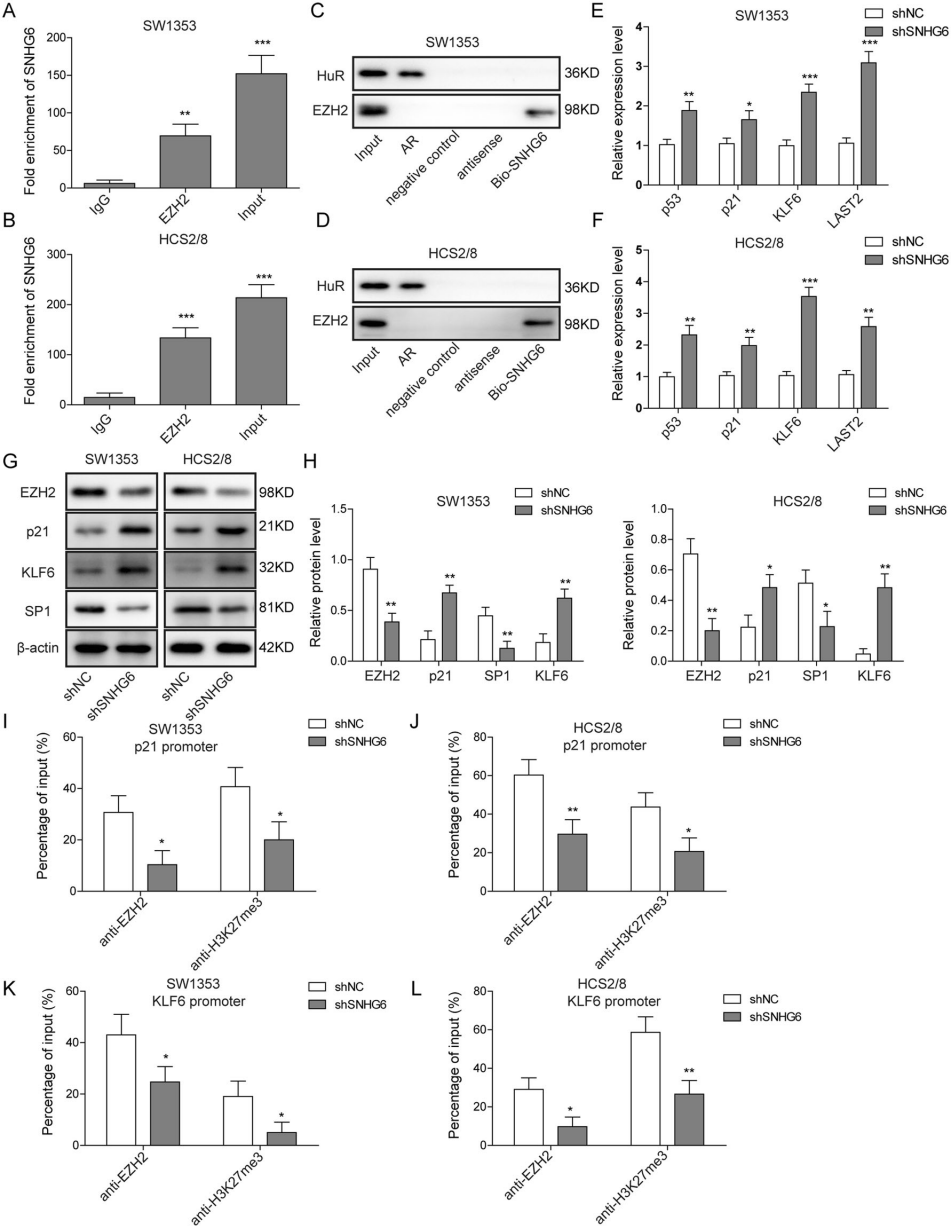
SNHG6 can restrain the expression of tumor-suppressor genes through direct interaction with EZH2 and the consequent histone methylation. **A**, **B** SW1353 (**A**) and HCS2/8 (**B**) cells were lysed and incubated with control IgG or anti-EZH2 antibody; the immunoprecipitate was subjected to qRT-PCR to measure the content of SNHG6. Cell lysate was used as the input control. **C**, **D** The interaction between SNHG6 and EZH2 was assessed by RNA pull-down assay in SW1353 (**C**) and HCS2/8 (**D**) cells, and the protein levels of HuR and EZH2 were examined by western blotting. 3′-Untranslated region (3′-UTR) of androgen receptor (AR) RNA was used as a positive control for HuR protein due to the UC-rich region of HuR in AR RNA; poly(A)_25_ RNA was employed as a negative control. **E**–**L** SW1353 and HCS2/8 cells were transfected with shNC or shSNHG6 by lentivirus, respectively. **E**, **F** The relative expression of p53, p21, KLF6, and LATS2 in SW1353 (**E**) and HCS2/8 (**F**) cells in the presence or absence of SNHG6 was determined by qRT-PCR. The mRNA levels of these four genes were normalized to GAPDH mRNA; experiments were performed in triple. **G**, **H** The expression of EZH2, p21, SP1, and KLF6 in SW1353 (**G**) and HCS2/8 (**H**) cells in the presence or absence of SNHG6 was measured by western blotting. β-Actin was used as the loading control. **I**–**L** The influence of SNHG6 knockdown on the binding of EZH2 to p21 promoter (**I**, **J**) or KLF6 promoter (**K**, **L**) and H3K27 methylation in p21 promoter (**I**, **J**) or KLF6 promoter (**K**, **L**) was examined by ChIP and qRT-PCR. **A**–**L** The data represented one of the three independent experiments. **A**, **B**, **E**, **F**, **H**–**L** Data are represented as mean ± SD. *p* values were determined by one-way analysis of variance (ANOVA) followed by Tukey post hoc test (**A**, **B**) or unpaired two-tailed *t* test (**E**, **F**, **H**–**L**). ***p < 0.001, **p < 0.01, *p < 0.05.

Previous study has revealed that SNHG6 could repress the transcription of p21 via recruiting EZH2 to the promoter region of p21 in colorectal cancer cells^14^; to further understand the underlying mechanism for the function of SNHG6 in chondrosarcoma cells, we first performed immunoprecipitation assay with control IgG and anti-EZH2 antibody using SW1353 and HCS2/8 cell lysates, and the immunoprecipitate was then subjected to qRT-PCR. Consistently, the data showed that SNHG6 could interact with EZH2 in chondrosarcoma cells (Fig. 3A, B). To confirm this finding, we then carried out RNA pull-down experiment with biotinylated SNHG6 or biotinylated antisense RNA in SW1353 and HCS2/8 cell lysates. The western blotting result clearly evinced that SNHG6 could pull down EZH2 in both SW1353 and HCS2/8 cells, not the control antisense RNA (Fig. 3C, D). Since EZH2 has been implicated in the regulation of multiple tumor-suppressor genes, we next determined the expression pattern of p53, p21, KLF6, and LATS2 in SNHG6-silenced SW1353 and HCS2/8 cells. The qRT-PCR results revealed that all these four genes were upregulated in chondrosarcoma cells when SNHG6 was knocked down (Fig. 3E, F). Moreover, we also performed western blotting experiment to assess the expression of EZH2, p21, Sp-1, and KLF6, and the data demonstrated that EZH2 and Sp-1 were downregulated while p21 and KLF6 were upregulated in SNHG6 knocked down cells (Fig. 3G, H). Consistently, the clinical correlation between the expression of EZH2, KLF-6, Sp-1, and SNHG6 in patient tumor samples also showed that both EZH2 and Sp-1 displayed the positive correlation with SNHG6, whereas KLF6 and p21 exhibited the negative correlation with SNHG6 (Fig. S2). To reveal the regulation mechanism of SNHG6 on p21 and KLF6, we next conducted the ChIP-qPCR assay with anti-EZH2 and anti-H3K27me3 antibodies in control or SNHG6-silenced SW1353 and HCS2/8 cells. Interestingly, the binding of EZH2 to the promoter region of p21 and KLF6 was impaired when SNHG6 was knocked down; moreover, the tri-methylation of histones (H3K27me3) in the promoter region of p21 and KLF6 was also reduced upon SNHG6 downregulation (Fig. 3I–L), which indicated that the repression of downstream genes was partially relieved. Taken together, our data here proved that SNHG6 could directly interact with EZH2 and repress the expression of tumor-suppressor genes through H3K27me3 histone methylation.

### SNHG6 is regulated by SP1 on transcriptional level and is involved in the tumor-promoting function of SP1

**Fig. 4:**
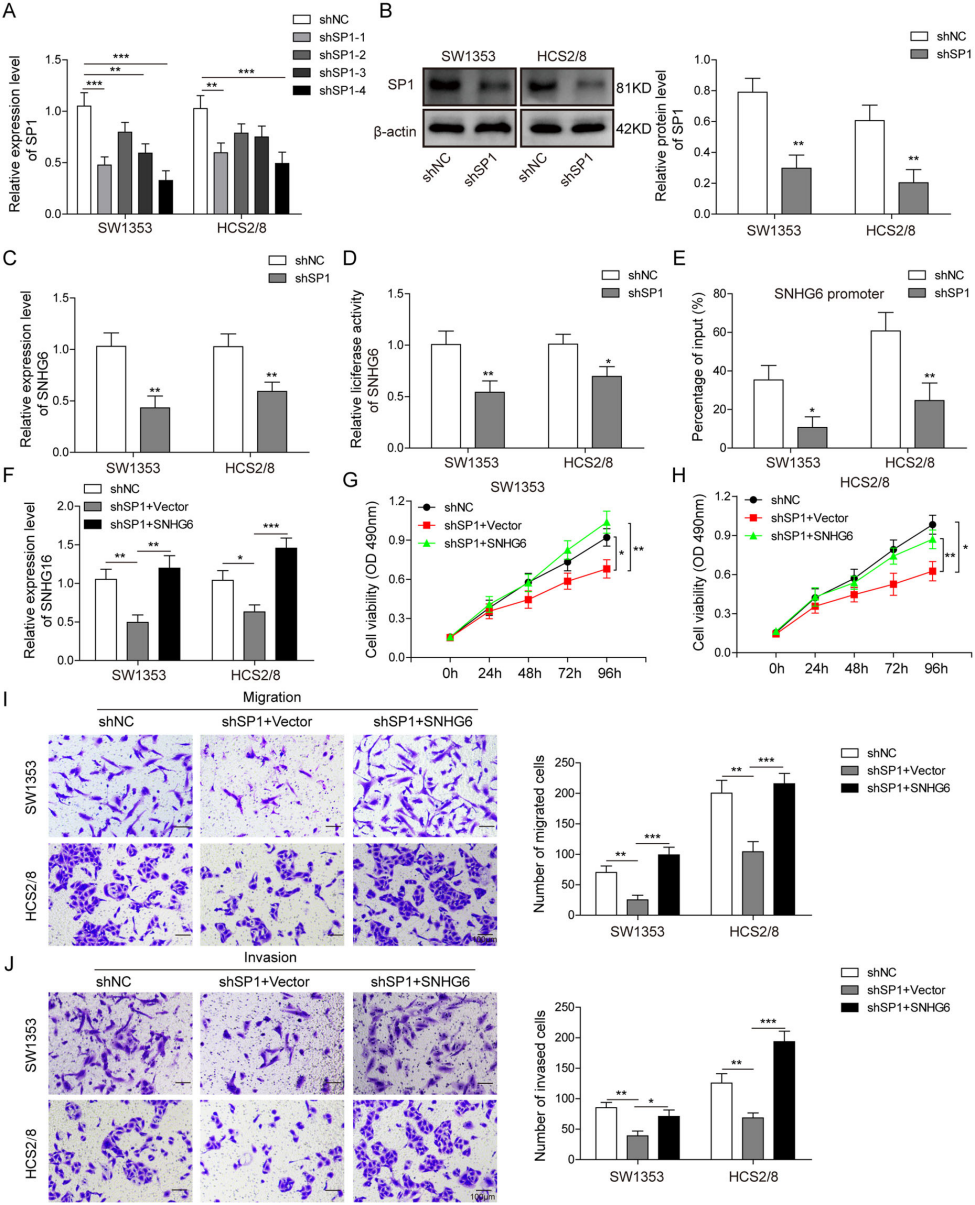
SNHG6 is regulated by SP1 on transcriptional level and is involved in the tumor-promoting function of SP1. **A** SW1353 and HCS2/8 cells were transfected with shNC or various shSP1s by lentivirus, respectively. The relative expression of SP1 in SW1353 and HCS2/8 cells was determined by qRT-PCR. The mRNA level of SP1 was normalized to GAPDH mRNA; experiments were performed in triple. **B**–**K** SW1353 and HCS2/8 cells were transfected with shNC or shSP1#4 by lentivirus, respectively. **B** The expression of SP1 was measured by western blotting. β-Actin was used as the loading control. **C** The influence of SP1 knockdown on the expression of SNHG6 was assessed by qRT-PCR. The SNHG6 RNA level was normalized to GAPDH RNA; experiments were performed in triple. **D** The influence of SP1 knockdown on the transcription of SNHG6 was measured by dual-luciferase assay. **E** ShNC- or shSP1-transfected SW1353 and HCS2/8 cells were then lysed and probed with anti-SP1 antibody; the precipitate was then analyzed by qRT-PCR to measure the level of SNHG6 promoter region. **F**–**I** SP1-silenced SW1353 and HCS2/8 cells were transfected with empty vector or SNHG6-overexpressing plasmid. **F** The expression of SNHG6 was analyzed by qRT-PCR. The SNHG6 RNA level was normalized to GAPDH RNA; experiments were performed in triple. **G**, **H** The proliferation of SW1353 (**G**) and HCS2/8 (**H**) cells was measured by MTT assay. **I**, **J** The migration and invasion of SW1353 (**I**) and HCS2/8 (**J**) cells were measured by transwell assay; the statistical results are shown on the right histogram. Scale bar, 50 μm. **A**, **C**–**J** Data are represented as mean ± SD. *p* values were determined by one-way analysis of variance (ANOVA) followed by Tukey post hoc test (**A**, **F**, **I**, **J**), unpaired two-tailed *t* test (**C**–**E**), or two-way ANOVA (**G**, **H**). ***p < 0.001, **p < 0.01, *p < 0.05.

Previous study has revealed that SP1 activation contributes to the high expression of SNHG6 in colorectal cancer^20^; to investigate whether SP1 also participated in the regulation of SNHG6 in chondrosarcoma cells, we then designed one control shRNA (shNC) and four shRNAs targeting SP1 (shSP1). After the transduction of these shRNAs into SW1353 and HCS2/8 cells, the knocking down efficiencies of four shRNAs were determined by qRT-PCR. From the data, we found that all four shRNAs showed various degree of knocking down effects, in which shSP1–4 exhibited the highest silencing efficiencies (Fig. 4A), which was thus used in the subsequent experiments. Western blotting experiment was also applied to confirm the knocking down effect of shSP1 on the protein level, and the result clearly demonstrated that SP1 expression was severely repressed (Fig. 4B). Next, we measured the relative expression of SNHG6 in control or SP1-silenced SW1353 and HCS2/8 cells by qRT-PCR, and the results illustrated that SP1 knockdown led to the downregulation of SNHG6 (Fig. 4C), which indicated that SP1 was required for the expression of SNHG6. To evince the interaction between SP1 and SNHG6, we cloned the promoter region of SP1 and inserted it into the upstream of firefly luciferase. This luciferase reporter plasmid was then co-transfected with *Renilla* luciferase control plasmid into SW1353 and HCS2/8 cells; the following dual-luciferase assay demonstrated that SP1 knockdown indeed decreased the luciferase activity, which suggested that SP1 could interact with the promoter of SNHG6 and control its expression (Fig. 4D). Moreover, we conducted ChIP-qPCR assay with anti-SP1 antibody in SW1353 and HCS2/8 cells, and SP1 knockdown evidently reduced the association between SP1 and the promoter region of SNHG6 (Fig. 4E). To counteract the influence of SP1 knockdown on SNHG6 expression, we then transfected SP1-silenced SW1353 and HCS2/8 cells with SNHG6-overexpressing plasmid or empty vector. The qRT-PCR results showed that the expression of SNHG6 was reinstated, even higher than that in control cells, after the ectopic expression of SNHG6 (Fig. 4F). Afterwards, we performed MTT assay, transwell migration, and invasion assay with SNHG6-rescued cells. Consistently, overexpression of SNHG6 in SP1-silenced cells completely rescued the impairment of cell proliferation, migration, and invasion due to SP1 knockdown (Fig. 4G–J). In summary, these data elucidated that SNHG6 was regulated by SP1 on the transcriptional level and was involved in the tumor-promoting effect of SP1.

### KLF6 expression is downregulated in chondrosarcoma

**Fig. 5:**
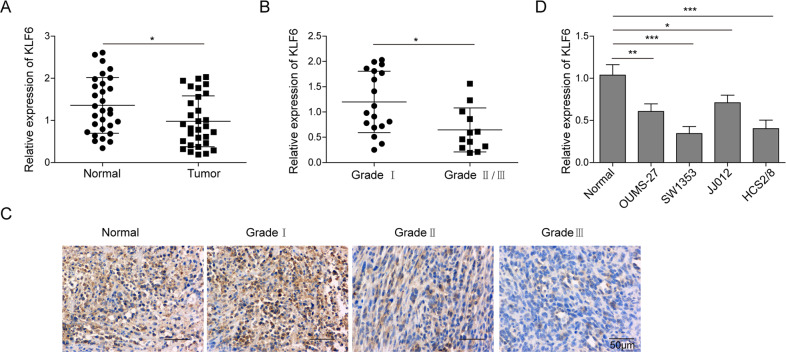
KLF6 expression is downregulated in chondrosarcoma. **A** The relative expression of KLF6 in normal cartilage tissues and chondrosarcoma tissues (*n* = 30) was assessed by qRT-PCR. **B** The relative expression of KLF6 in chondrosarcoma tissues from grade I (*n* = 18) and grade II/III (*n* = 12) were determined by qRT-PCR. **C** The expression of KLF6 in normal cartilage tissues and chondrosarcoma tissues from grade I to grade III was measured by IHC. Scale bar, 50 μm. **D** The relative expression of KLF6 in normal chondrocytes and various chondrosarcoma cell lines was measured by qRT-PCR. **A**, **B**, **D** The mRNA level of KLF6 was normalized to GAPDH mRNA; experiments were performed in triple. **A**–**D** The data represented one of the three independent experiments. **A**, **B**, **D** Data are represented as mean ± SD. *p* values were determined by unpaired two-tailed Student’s *t* test (**A**, **B**) or one-way ANOVA followed by Tukey post hoc test (**D**). ***p < 0.001, **p < 0.01, *p < 0.05.

As one crucial tumor suppressor, KLF6 has been found to bind to the promoter region of SP1 and reduce its expression in HCC^21^. To investigate the involvement of KLF6 in the progression of chondrosarcoma, we then measured the expression of KLF6 in normal tissues and tumor tissues of human chondrosarcoma by qRT-PCR. As expected, the expression of KLF6 was lower in tumor tissues than that in normal tissues (Fig. 5A). Meanwhile, we assessed the association between KLF6 expression and the progression of chondrosarcoma; the qRT-PCR data showed that the expression of KLF6 was even lower in tumor tissues in grade II/III than those in grade I, suggesting that KLF6 might be negatively associated with the progression of chondrosarcoma (Fig. 5B). Moreover, representative IHC images also confirmed this phenotype (Fig. 5C). At last, we examined the relative expression of KLF6 in normal chondrocytes and several available chondrosarcoma cell lines by qRT-PCR; consistently, KLF6 expression was reduced in all the chondrosarcoma cell lines, and SW1353 and HCS2/8 cells showed the least expression of KLF6 (Fig. 5D). Collectively, these experiments demonstrate that KLF6 expression was downregulated in chondrosarcoma.

### Overexpression of KLF6 represses the proliferation, migration, and invasion of chondrosarcoma cells

**Fig. 6:**
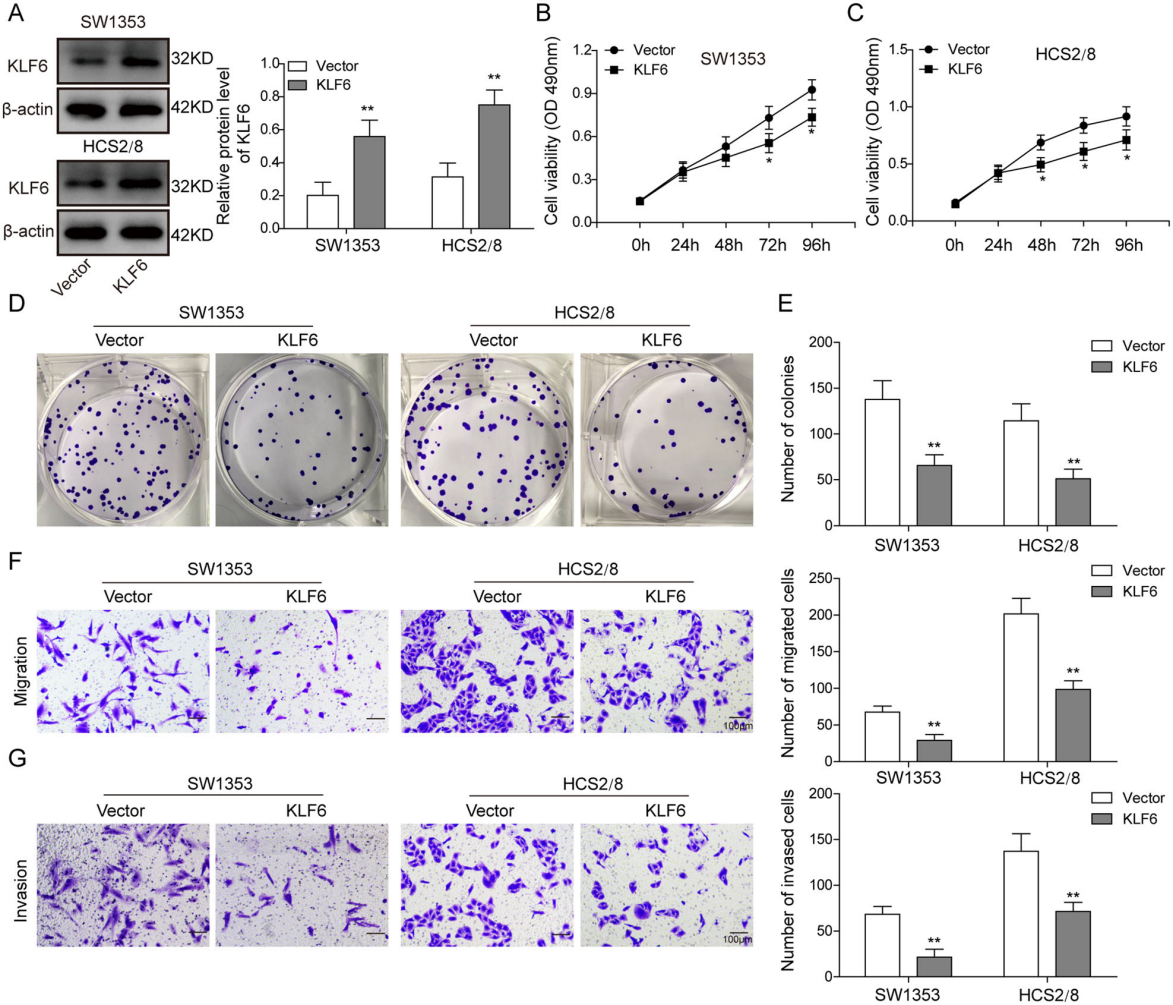
Overexpression of KLF6 represses the proliferation and migration of chondrosarcoma cells. **A**–**G** SW1353 and HCS2/8 cells were transfected with empty vector or KLF6-overexpressing plasmid. **A** The expression of KLF6 in SW1353 and HCS2/8 cells was measured by western blotting; β-actin was used as the loading control. **B**, **C** The proliferation of SW1353 (**B**) and HCS2/8 (**C**) cells was measured by MTT assay. **D** The influence of KLF6 overexpression on the proliferation of SW1353 and HCS2/8 cells was assessed by colony-formation assay. **E** The statistical results of colonies are shown in the right histograms. Scale bar, 5.0 mm. **F**, **G** The migration (**F**) and invasion (**G**) of SW1353 and HCS2/8 cells were measured by transwell assay; the statistical results are shown on the right histogram. Scale bar, 50 μm. **A**–**G** The data represented one of the three independent experiments. **B**, **C**, **E**–**G** Data are represented as mean ± SD. *p* values were determined by two-way ANOVA (**B**, **C**) or unpaired two-tailed Student’s *t* test (**E**–**G**). **p < 0.01, *p < 0.05.

To reinstate the expression of KLF6 in chondrosarcoma cells, we then overexpressed KLF6 in SW1353 and HCS2/8 cells by lentivirus, and western blotting experiment demonstrated that KLF6 expression was significantly upregulated (Fig. 6A). Afterwards, we performed MTT assay, colony-formation assay, transwell migration, and invasion assay to evaluate the influence of KLF6 overexpression on the proliferation, migration, and invasion of chondrosarcoma cells. As expected, KLF6 overexpression partially repressed the proliferation of SW1353 and HCS2/8 cells, especially at 72 and 96 h post the assay initiation (Fig. 6B, C). In cell colony-formation assay, KLF6 overexpression resulted in less colonies when compared with that in control cells (Fig. 6D, E). Furthermore, the migration and invasion capacities of chondrosarcoma cells were also impaired by KLF6 overexpression (Fig. 6F, G). Overall, these results substantiated that KLF6 overexpression could inhibit the proliferation, migration, and invasion of chondrosarcoma cells.

### KLF6 suppresses the expression of SNHG6 via inhibiting the transcription of SP1

**Fig. 7:**
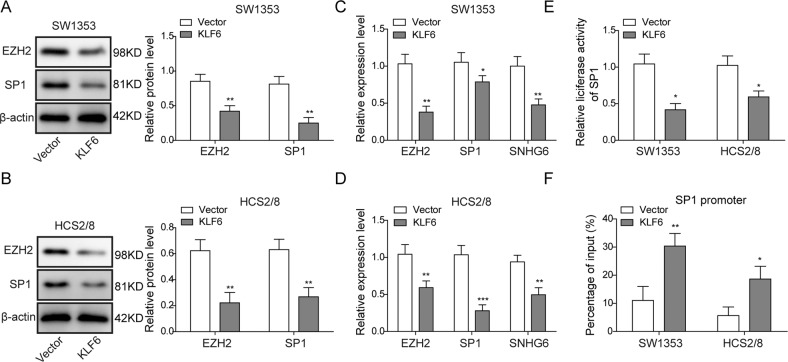
KLF6 suppresses the expression of SNHG6 via inhibiting the transcription of SP1. **A**–**F** SW1353 and HCS2/8 cells were transfected with empty vector or KLF6-overexpressing plasmid. **A**, **B** The expression of EZH2 and SP1 in SW1353 (**A**) and HCS2/8 (**B**) cells was measured by western blotting; β-actin was used as the loading control. The normalized protein expression level is shown in the right histograms. **C**, **D** The relative expression of EZH2, SP1, and SNHG6 in SW1353 (**C**) and HCS2/8 (**D**) cells was assessed by qRT-PCR. The mRNA levels of EZH2 and SP1 were normalized to GAPDH mRNA; SNHG6 RNA level was normalized to GAPDH RNA; experiments were performed in triple. **E** The influence of KLF6 overexpression on the transcription of SP1 was measured by dual-luciferase assay. **F** The binding of KLF6 to the promoter region of SP1 was assessed by ChIP and qRT-PCR experiments in both SW1353 and HCS2/8 cells. **A**–**F** The data represented one of the three independent experiments. **C**–**F** Data are represented as mean ± SD. *p* values were determined by unpaired two-tailed Student’s *t* test. ***p < 0.001, **p < 0.01, *p < 0.05.

To ascertain the association among KLF6, EZH2, SP1, and SNHG6 during the progression of chondrosarcoma, we then measured the expression of EZH2 and SP1 in KLF6-overexpressing SW1353 and HCS2/8 cells. Western blotting results displayed that both EZH2 and SP1 were downregulated upon KLF6 overexpression (Fig. 7A, B). Moreover, the qRT-PCR experiments manifested that the transcription of EZH2, SP1, and SNHG6 were all suppressed in the case of KLF6 overexpression (Fig. 7C, D). Since KLF6 has been reported as one suppressor of SP1, we thus designed luciferase reporting construct by cloning and inserting the promoter region of SP1 in the upstream of firefly luciferase. This reporter construct was then co-transfected with *Renilla* luciferase control plasmid into control or KLF6-overexpressing SW1353 and HCS2/8 cells; the following dual-luciferase assay demonstrated that KLF6 overexpression restrained the expression of SP1, indicated by the reduced luciferase activity, which suggested that KLF6 could interact with the promoter of SP1 and inhibit its expression (Fig. 7E). In addition, we implemented ChIP-qPCR assay using anti-KLF6 antibody in control or KLF6-overexpressing cells, and qPCR results reflected that there were more KLF6 binding to the promoter of SP1, further confirming that SP1 transcription was repressed in KLF6-overexpressing cells (Fig. 7F). Collectively, our results herein illustrated that KLF6 could hinder the transcription of SP1 and then restrain the expression of SNHG6.

### The tumor-promoting effect of SNHG6 partially depends on the repression of KLF6

**Fig. 8:**
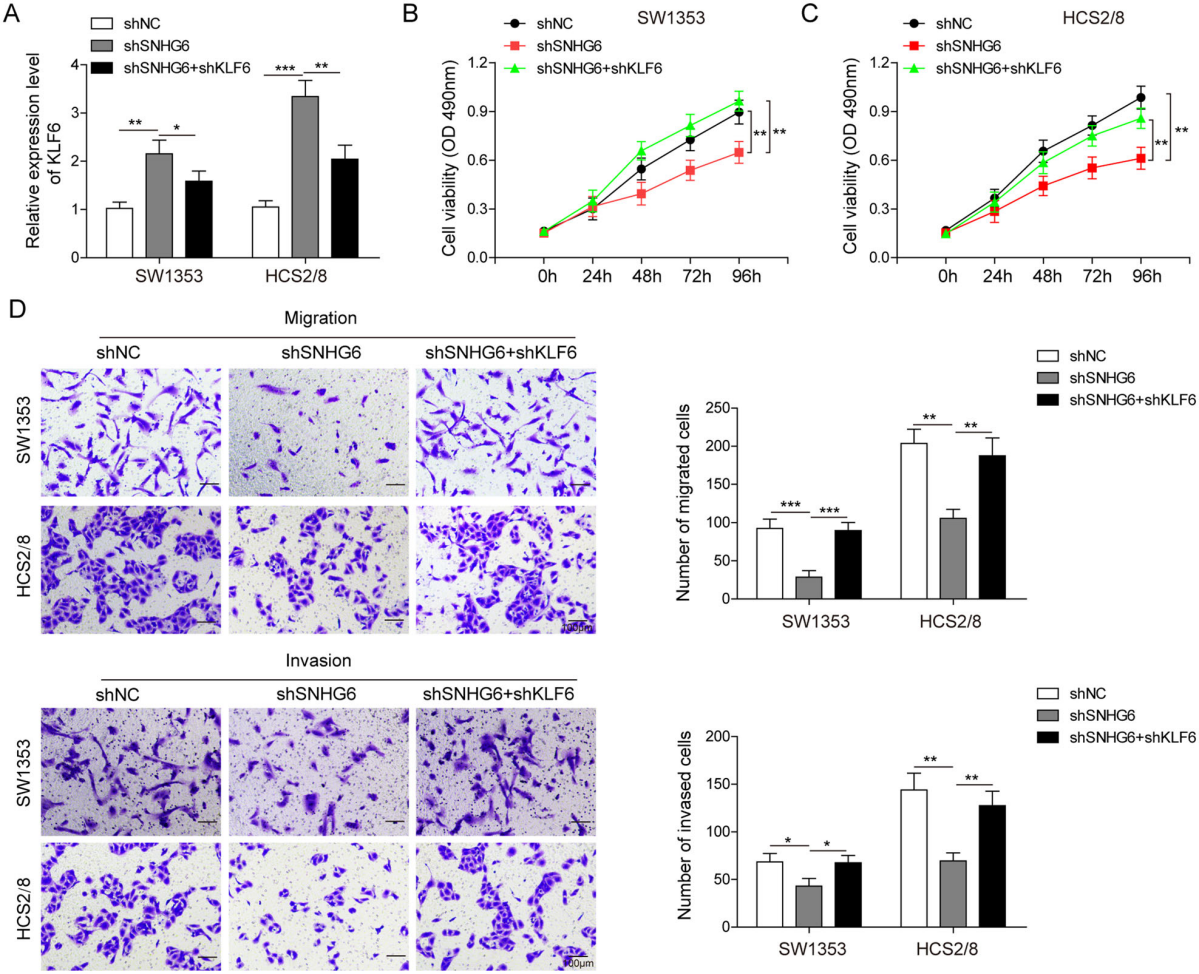
The pro-tumor effect of SNHG6 partially depends on the repression of KLF6. **A**–**E** SW1353 and HCS2/8 cells were transfected with shNC, shSNHG6, or shSNHG6 plus shKLF6 by lentivirus, respectively. **A** The relative expression of KLF6 in SW1353 and HCS2/8 cells was determined by qRT-PCR. The mRNA level of KLF6 was normalized to GAPDH mRNA; experiments were performed in triple. **B**, **C** The proliferation of SW1353 (**B**) and HCS2/8 (**C**) cells was measured by MTT assay. **D**, **E** The migration and invasion of SW1353 (**D**) and HCS2/8 (**E**) cells were measured by transwell assay; the statistical results are shown on the right histogram. Scale bar, 50 μm. **A**–**E** The data represented one of the three independent experiments. Data are represented as mean ± SD. *p* values were determined by one-way ANOVA followed by Tukey post hoc test (**A**, **D**, **E**) or two-way ANOVA (**B**, **C**). ***p < 0.001, **p < 0.01, *p < 0.05.

To pinpoint the sophisticated regulatory function of KLF6 on SNHG6 in the promotion of chondrosarcoma, we further knocked down KLF6 in both SW1353 and HCS2/8 cells by shRNA based on SNHG6 silencing. The qRT-PCR results showed that KLF6 expression was enhanced upon SNHG6 knockdown, whereas it was reduced with additional KLF6 knockdown (Fig. 8A). The subsequent MTT assay demonstrated that SNHG6 knockdown led to attenuated cell proliferation, while additional KLF6 knockdown completely rescued this phenotype (Fig. 8B, C). In the transwell migration and invasion assays, SNHG6 single knockdown obviously dampened the migration and invasion of SW1353 and HCS2/8 cells; however, SNHG6 and KLF6 double knockdown completely reinstated the migration and invasion capacities of SW1353 and HCS2/8 cells (Fig. 8D). In summary, these evidences manifested that the repression of KLF6 was indispensable for the tumor-promoting effect of SNHG6.

## Discussion

In the present study, we for the first time found that SP1 induced the upregulation of SNHG6, which then led to the suppression of KLF6 by recruiting EZH2 and promoted the tumorigenesis of chondrosarcoma. Although KLF6 could restrain the function of SP1, the repression of KLF6 by SNHG6 further increased the expression of SP1, which then continued to enhance SNHG6 expression as a positive feedback. This novel mechanism is the first report about the function of SNHG6 in chondrosarcoma and revealed that lncRNA SNHG6 and KLF6 played an essential role during the progression of chondrosarcoma (Fig. S3).

Several studies have investigated the tumor-promoting function of SNHG6 in the various tumors. In colorectal cancer, Yu et al. reported that SNHG6 was required for tumor cell survival, growth, migration, and invasion, which exerted its function through sponging miR-181a-5p and regulating E2F5 expression^22^. In osteosarcoma (OS), Xin Zhu and his colleagues found that knockdown of SNHG6 in OS could potently inhibit OS cell growth and proliferation, repress cell invasion, and induce cell apoptosis; mechanistic study revealed that SNHG6 sponged miR-26a-5p and thereby enhanced the expression of Unc-51-like autophagy activating kinase 1, leading to upregulated apoptosis by caspase 3 and autophagy by activating transcription factor 3^23^. In HCC, one report from Xiarong Hu’s group showed that SNHG6 acted as a competing endogenous RNA to sponge let-7c-5p, which thereby mediated the attenuation of c-Myc and the promotion of cell proliferation^24^. These works demonstrated that SNHG6 functioned as one oncogene through versatile pathways in distinct tumors. In our study, we found that SNHG6 recruited EZH2 to the promoter region of KLF6 and intensified the methylation, which thus repressed its transcription. This mechanism of SNHG6 was first reported in chondrosarcoma and was quite different from the general function of SNHG6 in early studies, since our results evinced that lncRNA could work as one epigenetic modulator to mediate the association between histone methyltransferase and target genes; however, this novel function required further investigation in other tumor cells. Moreover, we found SNHG6 also directly augmented the expression of EZH2, which was consistent with previous report on the association between SNHG6 and EZH2^20^.

SP1 is one crucial transcription factor implicated in the modulation of multiple cancers; specifically, SP1 regulates the expression of genes that are related with the cell proliferation and metastasis of various tumors. Thus SP1 is always regarded as one therapeutic target in chemotherapy^15^. Mechanistically, SP1 could directly bind to the promoter region of some pivotal proteins and suppress or augment their expression. For example, Hsu et al. found that SP1 expression was potently increased in early stage of lung cancer but declined in late stage, and the overexpression of SP1 in highly invasive lung cancer cells led to the upregulation of E-cadherin that suppressed the metastasis^25^; Yu and her colleagues reported that SP1 promoted the expression of FoxO3a after binding to its promoter region and thus enhanced the tumor progression of colorectal cancer^26^; Chuntao Tsai et al. found that SP1 suppressed the metastasis of chondrosarcoma via upregulation of tissue inhibitor of metalloproteinase-3^27^. In our present study, we identified that SP1 modulated the transcription of SNHG6, which demonstrated that SP1 could regulate the expression of tumor-associated proteins indirectly via lncRNAs. The similar function on lncRNAs has also been reported for other well-studied transcription factors, such as c-Myc and signal transducer and activator of transcription factor 3 (STAT3). Recently, Shaoxun Xiang et al found that c-Myc could repress the expression of lncRNA IDH1-AS1 and sustained the activation of Warburg effect by hypoxia-inducible factor 1α under normoxic conditions in cancer cells^28^; Hui Wang et al. reported that STAT3 could specifically bind to the promoter of HOXD-AS1 and activate the transcription of lncRNA HOXD-AS1, which was associated with the metastasis and invasion of HCC^29^. On the contrary, another interesting study from Hu’s laboratory found that SNHG6 could even upregulate the expression of c-Myc through sponging let-7c-5p in HCC^24^. The reciprocal regulation between other transcription factors and lncRNAs, especially SNHG6, in cancer progression suggested that there might be other transcription factors involved in the modulation of SNHG6 in chondrosarcoma.

Accumulative evidence has demonstrated the tumor-suppressing function of KLF6 in various tumors. For example, Peihao Wen and his colleagues found that KLF6 could suppress the proliferation and invasion of HCC cells both in vitro and in vivo^30^. In more detail, Ling-Min Kong et al. identified that KLF6 cooperated with SP1 to restrain the expression of basigin-2, which thus inhibited the proliferation, invasion, and metastasis of HCC^21^. In OS cells, KLF6 expression was significantly downregulated, which resulted in the suppression of viability, proliferation, and invasion of OS cells, whereas the apoptosis was enhanced^31^. In the present study, we for the first time revealed that KLF6 was downregulated and exerted tumor-suppressing function in chondrosarcoma. Mechanistical study demonstrated that KLF6 attenuated the expression of SP1, which was consistent with previous reports^21^. Another interesting finding was the positive feedback loop for SP1/SNHG6/EZH2/KLF6 axis, which severely repressed the expression and function of KLF6, one central regulator for the progression of chondrosarcoma; at the same time, the levels of SP1 and SNHG6 were further elevated due to the attenuation of KLF6. Both aspects contributed to the fast progression of chondrosarcoma in the end. Therefore, recovering the normal expression of KLF6 in tumor cells was one critical way to suppress the proliferation, migration, and invasion of chondrosarcoma.

In summary, our study revealed that the expression of SNHG6 was upregulated in chondrosarcoma, which was induced by SP1 activation. Mechanistically, augmented SNHG6 recruited EZH2 and inhibited the transcription of tumor-suppressor gene KLF6, which thus promoted the tumorigenesis of chondrosarcoma. Moreover, the inhibition of KLF6 by SNHG6 further enhanced SP1 expression and then induced SNHG6 expression to form a positive loop. This positive feedback loop potently facilitated the progression of chondrosarcoma both in vitro and in vivo. The novel mechanism revealed here not only elucidated the complex regulatory network behind chondrosarcoma but also might provide several potential therapeutic targets for the new treatment options of chondrosarcoma in future.

## Supplementary information

Supplementary material

FIGURE S1

FIGURE S2

FIGURE S3
