# Supplementary material for: SP1-induced long non-coding RNA SNHG6 facilitates the carcinogenesis of chondrosarcoma through inhibiting KLF6 by recruiting EZH2
Source: Cell Death Dis. 2021 Jan 11;12(1):59. doi: 10.1038/s41419-020-03352-6 (PMC7801621; doi:10.1038/s41419-020-03352-6)
Supplement: Supplementary file 1 — Supplementary material [file 41419_2020_3352_MOESM1_ESM.docx]

**Figure S1. The migration ability of normal chondrocytes and chondrosarcoma cell lines.**

The migration of normal chondrocytes and chondrosarcoma cell lines was determined by transwell assay. The results represented one of three independent experiments. Data were depicted as mean ± SD. *p* values were determined by one-way analysis of variance (ANOVA) followed by Tukey post hoc test. *** *p*< 0.001, ** *p*< 0.01, * *p*< 0.05.

**Figure S2. The clinical correlation between EZH2, KLF6, Sp-1 and SNHG6 in the progression of chondrosarcoma.**

(A) The correlation between SNHG6 expression and KLF-6 expression in patient tumor samples. n =30. (B) The correlation between SNHG-2 expression in patient tumor samples. n =30. (C) The correlation between SNHG6 expression and Sp-1 expression in patient tumor samples. n =30. (D) The correlation between SNHG6 expression and EZH2 expression in patient tumor samples. n =30. Data were represented as mean ± SD. *p* values were determined by unpaired two-tailed students’ *t*-test. *** *p*< 0.001, ** *p*< 0.01, * *p*< 0.05*.*

### Figure S3. A schematic diagram for the regulatory mechanism of SNGH6 in the progression of chondrosarcoma.
