# Supplementary figures and images for: SP1-induced long non-coding RNA SNHG6 facilitates the carcinogenesis of chondrosarcoma through inhibiting KLF6 by recruiting EZH2
Source: Cell Death Dis. 2021 Jan 11;12(1):59. doi: 10.1038/s41419-020-03352-6 (PMC7801621; doi:10.1038/s41419-020-03352-6)

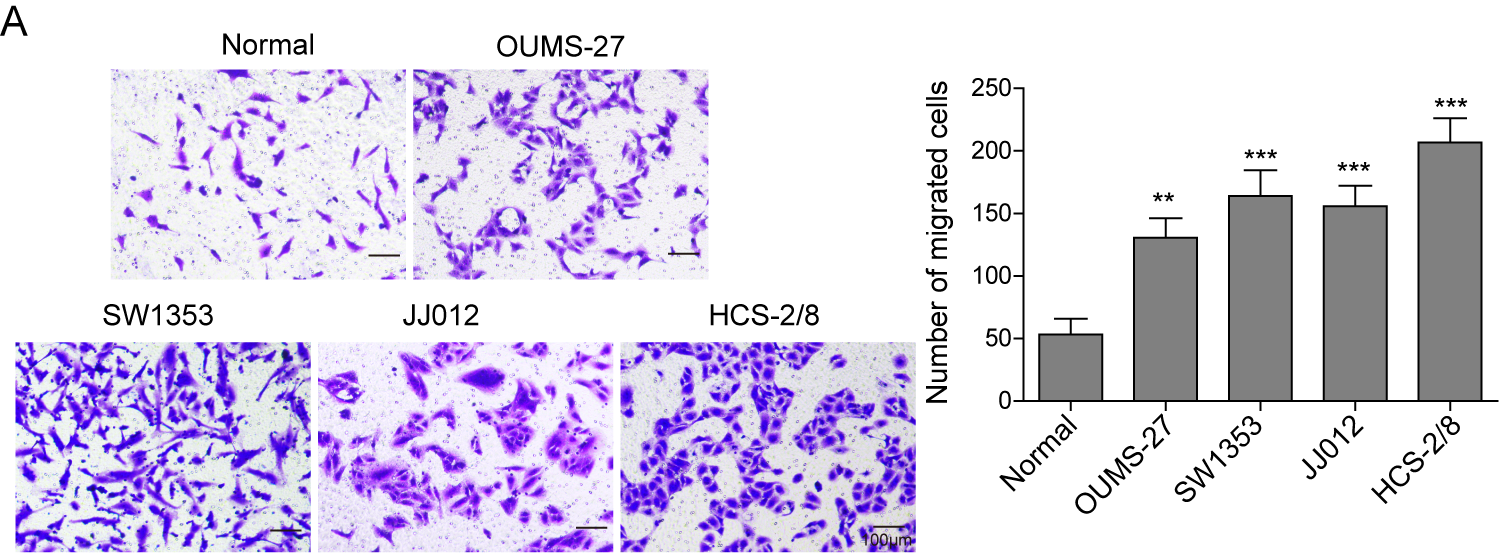

Supplement: Supplementary file 2 — FIGURE S1 [file 41419_2020_3352_MOESM2_ESM.tif]

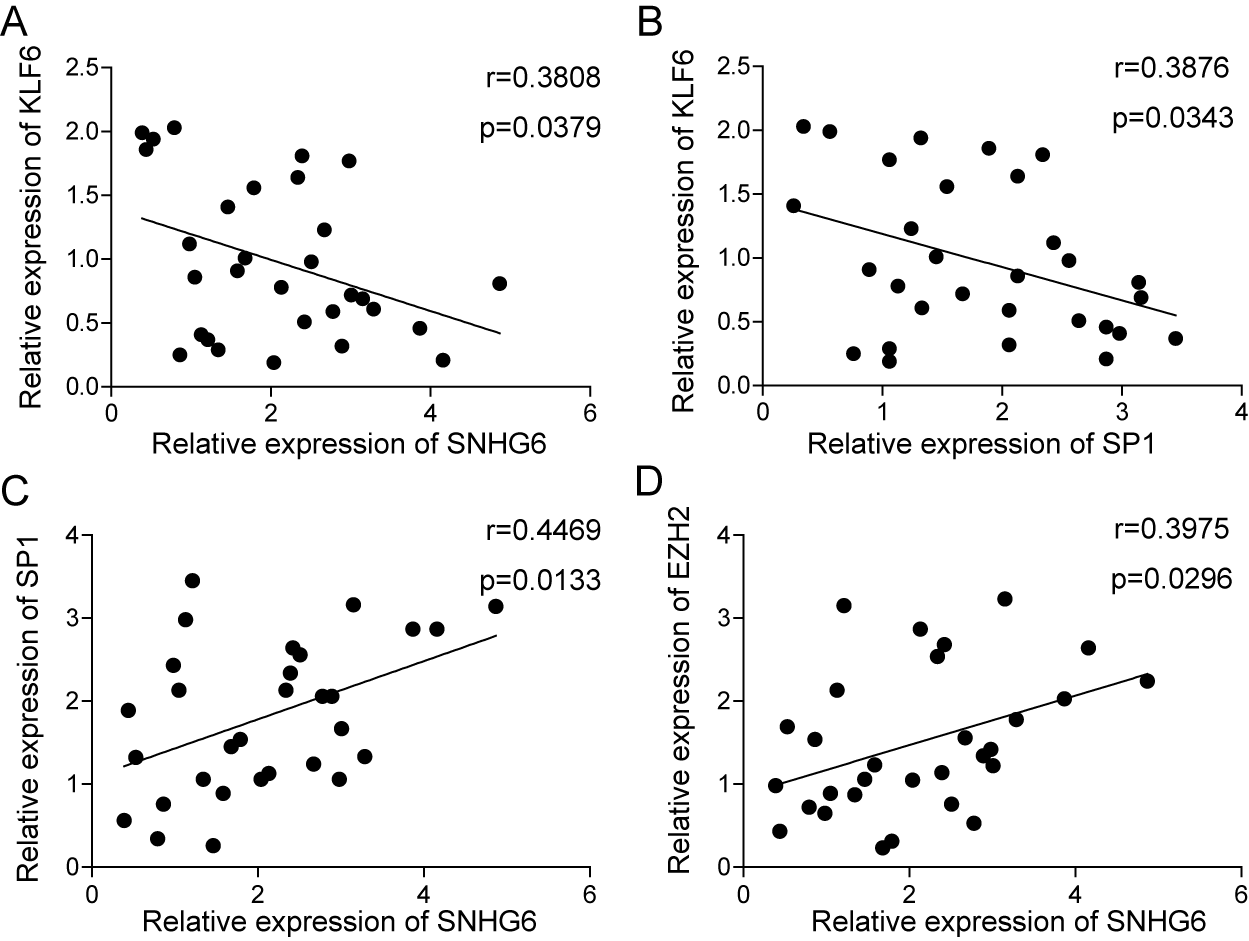

Supplement: Supplementary file 3 — FIGURE S2 [file 41419_2020_3352_MOESM3_ESM.tif]

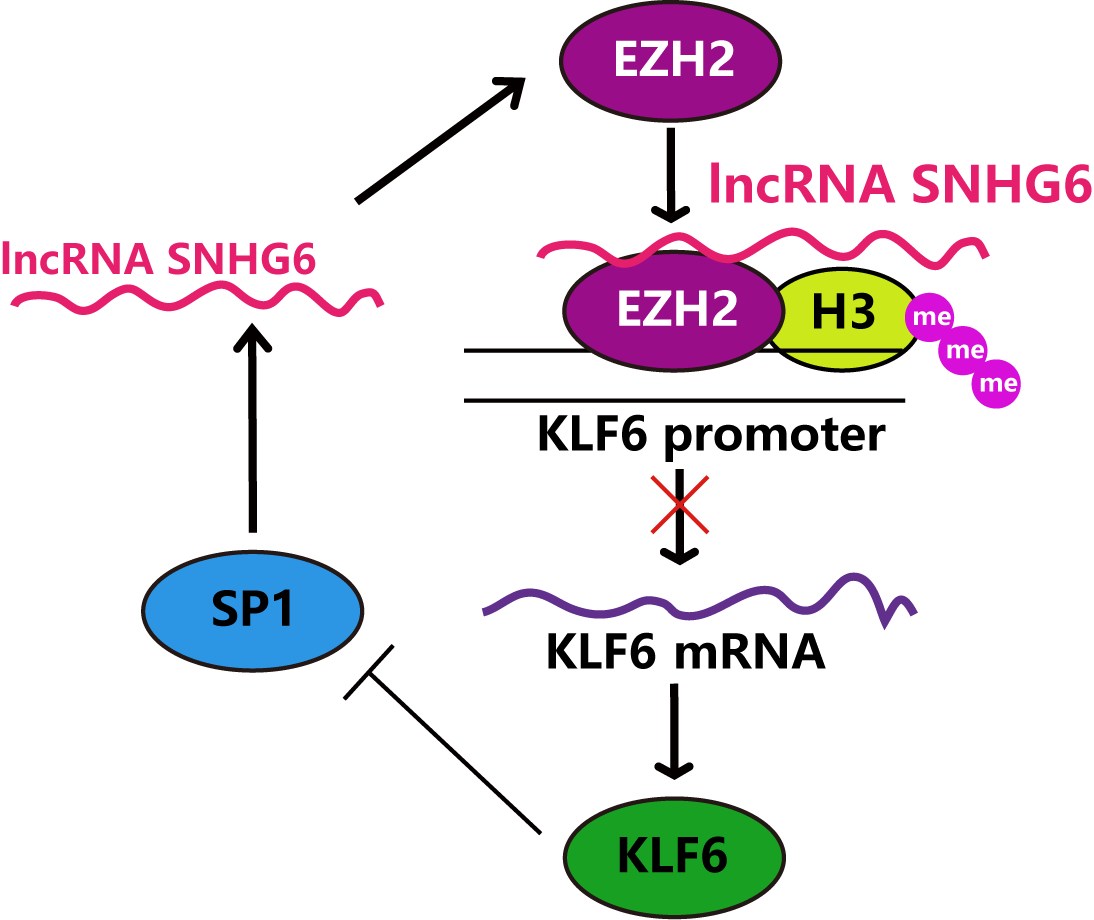

Supplement: Supplementary file 4 — FIGURE S3 [file 41419_2020_3352_MOESM4_ESM.tif]
